# Stable Isotope Geochemistry of the Organic Elements within Shales and Crude Oils: A Comprehensive Review

**DOI:** 10.3390/molecules27010034

**Published:** 2021-12-22

**Authors:** Abiodun Busuyi Ogbesejana, Bo Liu, Mehdi Ostadhassan

**Affiliations:** 1 State Key Laboratory of Shale Oil and Gas Enrichment Mechanism and Effective Development, Beijing 100101, China; aogbesejana@fudutsinma.edu.ng (A.B.O.); mehdi.ostadhassan@nepu.edu.cn (M.O.); 2 Institute of Unconventional Oil & Gas, Northeast Petroleum University, Daqing 163318, China; 3 Department of Applied Chemistry, Federal University Dutsin-Ma, Dutsin-Ma P.M.B. 5001, Nigeria

**Keywords:** stable isotopes, shales, crude oils, organic elements, hydrocarbon exploration

## Abstract

Over time, stable isotopes have proven to be a useful tool in petroleum geochemistry. However, there is currently insufficient literature on stable isotope geochemistry of the organic elements within shales and crude oils in many petroleum systems around the world. As a result, this paper critically reviews the early and recent trends in stable isotope geochemistry of organic elements in shales and crude oils. The bulk and compound-specific stable isotopes of H, C, and S, as well as their uses as source facies, depositional environments, thermal maturity, geological age, and oil–oil and oil–source rock correlation studies, are all taken into account. The applications of the stable isotopes of H and C in gas exploration are also discussed. Then, the experimental and instrumental approaches to the stable isotopes of H, C, and S, are discussed.

## 1. Introduction

The analysis of stable isotopes of various elements present in the organic matter (OM) enables us to understand diverse geochemical processes that take place during geological periods [1]. Stable isotopes have been used in gas and petroleum exploration for correlation [1,2,3,4,5,6,7,8], maturation [9,10,11,12], and OM evolution studies [1,2,3,4,5,6,7,8,9,10,11,12,13,14,15,16,17,18,19], as well as assessing the depositional environment of the source rocks [13,14,15,16,17,18,19]. Because OM is made up mostly of carbon (C) and hydrogen (H), with other heteroatoms, i.e., nitrogen (N), oxygen (O), and sulfur (S), knowing the stable isotopes of these elements is crucial for petroleum exploration [1]. However, the analysis of isotopic compositions of oxygen and nitrogen has been rarely employed in petroleum exploration, mainly due to analytical challenges in quantitatively converting organic oxygen and nitrogen to CO_2_ and N_2_ [1].

To understand geochemical and geological processes, stable isotope geochemistry uses the ratios of isotopes between distinct phases or chemical species in a compound [20]. Isotopes of an element are atoms with the same number of protons but a different number of neutrons, resulting in a different atomic mass [20]. In contrast to unstable or radioactive isotopes, stable isotopes have nuclei that do not decay over time. Thus, except for physical, chemical, and biological processes that lead to their fractionation, stable isotope abundances in geologic materials remain constant across time [20]. The ratio of two stable isotopes of the same element can be used to calculate the relative amounts of light and heavy isotopes in a phase or chemical species. The degree to which the light or heavy isotope is preferentially integrated into a substance during a phase transition or chemical reaction can be determined by comparing isotope ratios of materials [1,20]. Although, temperature, reaction kinetics, and mass can all influence the degree of this preferential incorporation, knowing such ratios can provide us with insight into reaction mechanisms, formation temperatures, and other information regarding the evolving earth [1,20,21]. 

C and H stable isotopes, since they are the most abundant elements, are important for determining the processes that sedimentary organic matter has undergone. Carbon and hydrogen, for example, both have two stable isotopes: ^12^C and ^13^C and ^1^H and ^2^D. ^12^C makes up to 98.899 wt.% of the whole carbon pool, while ^13^C makes up only 1.111 wt.% [22]. The complete hydrogen pool is made up of 99.985 wt% of ^1^H and 0.0105 wt% of ^2^D [22]. Silverman et al. [2], conducted the first in-depth isotopic investigation of petroleum. The initial basic understanding of petroleum isotope geochemistry was developed by [3,4,5,23,24], in the United States, [25,26] in East Germany, [27] in Italy, [28] in West Germany, and [6,29,30], in the Soviet Union. In addition, in the 1960s, the Galimov group at Gubkin’s Institute conducted theoretical and practical research of isotope fractionation in hydrocarbon systems, as well as oil and gas studies in the Volga–Ural, Sakhalin, and Kaspian regions, which are some of the most pioneering studies in this field reported in the literature [31,32,33,34,35,36,37,38,39,40].

Sulfur is a common element in nature that plays a key role in a variety of natural processes, and understanding sulfur isotopic composition sheds light on a wide range of geological events throughout history [41,42,43]. Sulfate is an important link between the carbon, sulfur, and iron cycles, particularly in sedimentary basins, through geochemical processes such as bacterial sulfate reduction (BSR), iron sulfide production, and organic matter sulfation [7,8,44,45]. Bacterial sulfate reduction (BSR) is an anaerobic process that occurs in anoxic conditions with abundant organic matter as a reductant and results in a depletion of ^34^S in sulfide products (such as pyrite) compared to coeval carbonate-associated sulfate if no obvious alteration occurs in the burial process [46,47,48].

Even though there are many papers on the stable isotope geochemistry of C, H, and S and their applications to hydrocarbon explorations, the literature is still insufficient when considering the importance of C, H, and S isotopes in oil and gas explorations. As a result, the early and modern uses of the stable isotope geochemistry of organic elements (C, H, S) within shales and crude oils are further explored in this review paper. 

## 2. Standards and Notation 

The stable isotope delta (δ) notation, which is expressed by the following equation, is the measured values relative to an internationally accepted standard.

δ(‰) = [(R_sample_ − R_standard_)/R_standard_] × 1000 (1)

R depicts the isotope abundance ratio, such as ^13^C/^12^C, ^18^O/^16^O, ^34^S/^32^S, ^15^N/^14^N, or D/H (^2^H/^1^H). For example, the δ value for carbon is a convenient way to characterize tiny differences in the relative abundance of ^13^C in biological matter. In comparison to the standard, a negative value indicates that the sample is depleted in the heavy isotope, whereas the positive value indicates that the sample is enriched in the heavy isotope. To express relative isotope composition, the terms “light” and “heavy” are replaced for carbon, and instead “^13^C-depleted” and “^13^C-enriched” are used, respectively. The isotopic ratios of stable isotopes and their corresponding reference standard are shown in Table 1 [21].

## 3. Stable Isotope Fractionation

Isotope fractionation is a physical phenomenon that occurs when mass differences between isotopes produce variations in relative abundance [49]. Isotopic fractionation occurs in nature as a result of binding strength during chemical, biological, and physical processes [20]. Isotopes that are “heavy” make stronger bonds than isotopes that are “light”. As a result of isotopic fractionation associated with these processes, the isotopic composition of crude oils and sediments, as well as their constituents, can be affected by isotopic source signatures and processes such as maturation and biodegradation [50,51,52]. Kinetics and equilibrium are the two fractionation processes that cause these isotopic shifts. While both processes may be active, the kinetic isotope impact is the most relevant in most hydrocarbon exploration investigations, such as in natural gas exploration studies [52,53,54].

### Kinetic Isotope Effect (KIE)

Different reaction rates for heavy vs. light isotopes would cause KIE related to degradation and maturation processes (e.g., ^13^C vs. ^12^C). The presence of a “heavy” species (e.g., ^13^C) in the degradation-targeted bond raises the bond cleavage activation energy and, as a result, slows the rate of degradation. The leftover substrate becomes increasingly enriched in “heavier” isotope species as degradation progresses (i.e., ^13^C/^12^C increases). Isotope effects associated with degradation are significant for the atoms present when the chemical bond is broken (primary isotope effects), whereas secondary isotope effects are moderate for the remaining atoms. Considering hydrogen, only secondary isotope effects are significant at the normal precision of CSIA (compound-specific isotopic analysis). When a biodegradation process exclusively targets a single atomic position (or positions) (e.g., oxidation of a methyl group in toluene), the overall expression of isotope fractionation, as assessed by CSIA, is “diluted”. The intrinsic role of an isotope that affects the atoms directly involved in the reaction is unaffected by molecule size and can be determined using CSIA data [20,21,37,38,55]. The initial rate-limiting phase of the reaction cycle is reflected by isotope effects on biodegradation. In other circumstances, the delayed step may not be a bond cleavage but rather a non-isotope fractionating process, such as the development of the substrate–enzyme complex. There will be no isotope fractionation if the latter is entirely rate limiting. The rule of thumb is that biodegradation isotope effects are restricted to tiny compounds that can easily pass through degraders’ cell membranes. This would include chlorinated ethenes and ethanes, methyl tertiary butyl ether (MTBE), and mono-aromatic compounds that have been extensively researched and where isotope fractionation has been seen [55,56]. Fractionation has been seen in the lighter hydrocarbons in the C_5_ to C_10_ range in exploration studies [57]. Semi-volatile environmental pollutants including long-chain n-alkanes, multi-ring polycyclic aromatic hydrocarbons (PAHs), and polychlorinated biphenyls (PCBs) often do not produce measurable isotopic fractionation during biodegradation. It is also worth noting that in larger compounds with more than 10–12 carbon atoms, the isotopic alterations that occur to the one carbon atom that is being attacked are diluted by the presence of the other carbon atoms that are not impacted. As a result, overall changes in the isotopic makeup of these bigger molecules are usually undetectable when isotope values are measured [21]. Isotope fractionation can also be caused by physical processes such as phase transitions, sorption, and diffusion [58,59,60]. Different kinetic energies of gaseous phase molecules result from the mass difference between isotope species, resulting in differential rates of vapor migration and different bond energies of light isotope- vs. heavy isotope-substituted molecules, affecting phase partitioning equilibria and evaporation–condensation [20,21]

## 4. Experimental and Instrumental Methods in Stable Isotopes (C, H, and S)

The analytical methods applied in the analyses of stable carbon, hydrogen, and sulfur isotopes are reported below.

### 4.1. Stable Isotopic Analyses

#### 4.1.1. Fractionation of Sediment Extracts and Crude Oils

Column chromatography with silica gel–alumina as the stationary phase is generally used to fractionate extracts and crude oils. A typical glass column is 50 cm long and has an internal diameter of 0.5 cm. The column is rinsed twice, with DCM first and then light petroleum spirit (petroleum ether). After that, the column is filled with n-hexane and cotton wool is used as a resting pad for the stationary phase, silica gel (SiO_2_). After that, the stationary phase (SiO_2_) is added. Two (2 g) of Alumina (Al_2_O_3_) is used to stabilize the surface. A total of 70 mL of n-hexane, 70 mL of dichloromethane–n-hexane (2:1, *v*/*v*), and 70 mL of dichloromethane–methanol (1:1, *v*/*v*) are used to elute the saturated, aromatic hydrocarbon, and polar fractions, respectively. Each fraction is recovered by carefully evaporating solvents on a rotary evaporator, followed by the removal of the leftover solvent under a nitrogen gas stream (modified from [61]). The recovered saturated hydrocarbon, aromatic hydrocarbon, and polar fractions can now be analyzed using elemental analysis–isotope ratio mass spectrometry (EA–irMS) (bulk isotope analysis). 

#### 4.1.2. Molecular Sieving

Straight chain hydrocarbons are commonly separated from branched and cyclic hydrocarbons by treating saturated fractions with activated (250 °C, 8 h) 5A molecular sieve [62] in cyclohexane. The n-alkanes are recovered by dissolving the filter in HF (2 mL, 50% *w*/*v*) and neutralizing the solution with saturated sodium bicarbonate [9]. n-pentane is used to extract the aqueous phase (1 mL × 5). This fraction has n-alkanes that could be analyzed using gas chromatography–isotope ratio mass spectrometry (GC–irMS). Using a ZSM-5 molecular sieve, the branched and cyclic (5A excluded) hydrocarbon fractions from crude oils and sediments might be further simplified by removing methyl alkanes and alkyl cyclohexanes. The branched or cyclic (5A excluded) fraction is transferred to a small column (7–8 cm × 0.5 cm i.d.) of activated (250 °C) ZSM-5 molecular sieve (pre-rinsed with pentane) and allowed to stand (1–5 min). The branched or cyclic fraction is obtained after rinsing the filter with pentane (2 mL) (ZSM-5 excluded) [10]. 

#### 4.1.3. Elemental Analysis–Isotope Ratio Mass Spectrometry (Bulk δ^13^C Analysis)

The principle behind determining the isotopic composition of individual molecules or the bulk stable isotope values of complicated mixtures is fairly similar. Regardless of the sample’s origin, each process necessitates complete combustion and conversion to CO_2_ and water [21]. For bulk determinations, the sample can be converted to CO_2_ using an elemental analyzer interfaced directly to an isotope ratio mass spectrometer in a sealed tube in the presence of CuO, or the sample can be converted to CO_2_ using an elemental analyzer interfaced directly to an isotope ratio mass spectrometer. A stable isotope ratio mass spectrometer is used to transfer and analyze CO_2_, which is made up of both ^13^CO_2_ and ^12^CO_2_. The relative amounts of ^13^CO_2_ and ^12^CO_2_ are measured in comparison to a standard material, Vienna Pee Dee belemnite (VPDB), which has a carbon isotope value of 0, given in the notation (see Table 1). Almost all samples will have lower ^13^C levels than the standard and will be depleted in ^13^C, resulting in negative ^13^C results. Stable carbon isotope ratios (R = ^13^C/^12^C) are expressed relative to the VPDB standard using the “delta” notation, where: δ ^13^C = (R_sample_/R_standard_ − 1) × 1000 (units are‰ or per mil or parts per thousand) [21]. The sample is weighed inside a small silver capsule, which is then folded and dropped into a pyrolysis reactor containing glassy carbon chips kept at 1260 °C for bulk D analysis. The sample is pyrolyzed to produce H_2_ and CO, as well as N_2_ if necessary. The pyrolysis products are separated on a 1 m chromatographic column packed with 5A molecular sieve and kept at 80 °C in an oven before being passed via a thermal conductivity detector (TCD) and into the irMS instrument. The D values are calculated and expressed in delta notation to VSMOW (Vienna standard mean ocean water) [10]. By precipitating barium sulfate, which is then combusted to produce sulfur dioxide, a custom-built stable isotope ratio mass spectrometer [63] can perform stable sulfur isotope ratio analysis. The sulfur isotopes are expressed in terms of the CDT standard. The ratios of the other elements, such as O, N, or Cl, are represented in the same way with their unique standard. Table 1 shows the natural abundance of the stable isotopes typically employed in geochemistry, as well as the international standards used to calculate their values. 

#### 4.1.4. Gas Chromatography–Isotope Ratio Mass Spectrometry (GC-IRMS)

**Figure 1:**
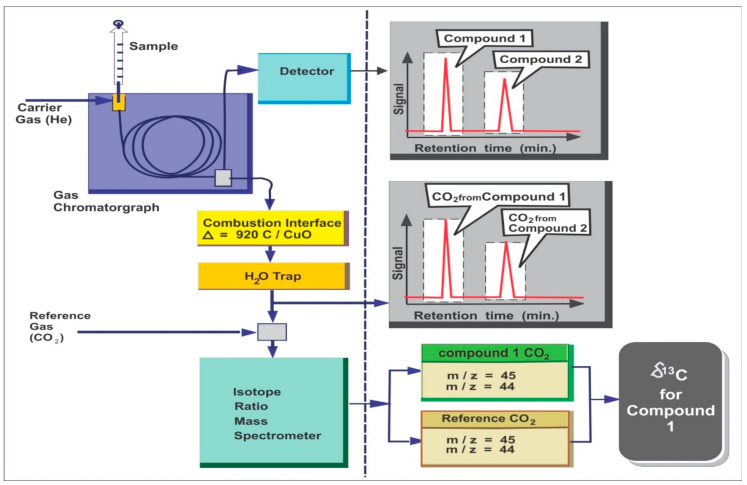
Schematic diagram of GC-IRMS system (modified from [21]).

A gas chromatograph coupled to the combustion system can allow the carbon isotope ratios to be determined on individual organic compounds (GC/combustion/IRMS, also called compound-specific isotope analysis (CSIA). After being eluted from the GC column, the components pass through a combustion reactor and are combusted to CO_2_ and water to determine the isotopic composition of individual compounds using GC-IRMS (Figure 1). The water is removed, and the CO_2_ is transferred to an isotope ratio mass spectrometer, which determines the relative amounts of ^13^CO_2_ and ^12^CO_2_ and calculates ^13^C values for each molecule. A Micromass IsoPrime isotope ratio mass spectrometer interfaced to a gas chromatograph for determining compound-specific stable carbon isotopic composition (^13^C values) or a chromatograph for determining stable hydrogen isotopic compositions (D values) is commonly used in GC–irMS [10]. For routine analysis, the oven is set to a temperature range of 40 to 310 °C at a rate of 3 °C/min, with initial and final hold times of 1 and 30 min, respectively. Samples are injected into an autosampler utilizing a split or splitless injector in pulsed splitless mode. Ultra-high purity (UHP) He is commonly utilized as carrier gas with a flow rate of 1.1 mL/min and a constant flow injector. The aromatic fractions of the samples are separated by a GC coupled to an inductively coupled plasma mass spectrometer to determine the sulfur isotope ratios of individual dibenzothiophenes. The operating conditions are described in detail in [64,65,66]. The samples are run in duplicates to ensure accuracy and the standard deviation between the two duplicates is noted accordingly [11]. The schematic diagram of GC-IRMS is shown in Figure 1 [21].

#### 4.1.5. Methods of Stable Sulfur Isotope Analysis

SO_2_ is the gas of choice for gas–source mass spectrometric measurements. With the emergence of online combustion technologies [67], multistep offline preparations have been simplified to a single step, namely combustion in an elemental analyzer. Sample preparations have become less time-consuming and less reliant on potentially fractionating wet chemical extraction procedures, resulting in a minimum sample gas of less than 1 mg. Puchel et al. [68] and Rees [69] initially described a method that uses SF_6_ instead of SO_2_, which has several advantages, including no mass spectrometer memory effect and no need for raw data corrections of recorded isotope ratios because fluorine is monoisotopic. The reliability of the SO_2_ correction for oxygen isobaric interferences has been called into doubt by a comparison of ^34^S-values produced using the traditional SO_2_ and the laser SF_6_ techniques [70]. As a result, despite the high toxicity of the gases, the SF_6_ method has been revived [71], revealing that SF_6_ is an appropriate gas for detecting ^33^S/^32^S, ^34^S/^32^S, and ^36^S/^32^S ratios. Sulfur isotope ratios can now be determined using microanalytical techniques such as laser microprobe [71,72,73,74] and ion microprobe [75,76,77,78,79]. Hauri et al. [80] used NanoSIMS to collect data on several sulfur isotopes.

#### Bulk Stable Sulfur Isotope Analysis

Cai et al. [12] described the procedures for separating kerogen and analyzing sulfur isotopes in kerogen and oil. Pyrite is usually removed from kerogen by introducing a mixture of hot 6N HCl and CrCl_2_ to the ground dry kerogen under a nitrogen flow, with the H_2_S being carried to a trap and recovered as Ag_2_S. Water washing removes excess acids and acid-soluble ions from residual kerogen. The residual kerogen is collected and reground 2 h later to expose additional pyrite surfaces, and the process is repeated. Following the two treatments, the leftover kerogen is examined using X-ray diffraction (XRD) to see if pyrite levels are below detection limits (60.5% depending on conditions). If not, more treatments would be necessary. To oxidize organically linked sulfides to sulfate, a known weight (between 350 mg and 900 mg) of kerogen isolate or 1–4 g of oil is combusted in a Parr bomb apparatus at 25 atm oxygen. Total residual kerogen sulfur is calculated by precipitating dissolved sulfate as BaSO_4_ and weighing it. The maximum residual pyrite content in the kerogen after the chromium reduction is determined by measuring dissolved iron at pH < 2 with an atomic absorption spectrometer (assuming that all Fe occurs as pyrite in the kerogen). The produced BaSO_4_ is only evaluated for δ^34^S when leftover kerogen samples have contaminated pyrite sulfur and total sulfur < 0.08 to ensure low levels of errors (based on the differences in ^34^S value between kerogen and the related pyrite).

#### Individual Sulfur Compounds Isotope Analysis

Bendall et al. [81], Craddock et al. [82], and Parris et al. [83] have reported the use of multi-collector–inductively coupled plasma mass spectrometer (MC-ICP-MS) techniques. Amrani et al. [64] proposed a method for analyzing individual sulfur organic compounds using a gas chromatography–multi-collector–inductively coupled mass spectrometer (GC-MC-ICP-MS). Sample sizes are at orders of magnitude smaller than what is commonly used for SO_2_ and SF6 [84] due to poor detection limits. The GC-MC-ICP-MS method does not require any chemical preparation and allows for simultaneous collection of all four sulfur isotopes; however, it has lesser precision than other methods. The aromatic fractions of the oils and sediment extracts are separated by GC-MC-ICPMS to determine the sulfur isotope ratios of specific sulfur compounds. Amrani et al. [64,65] and Said-Ahmad and Amrani [66] described the system and its working conditions in detail. Duplicates for some of the oils and sediment extracts are measured and the standard deviation between the two duplicates is usually better than 1‰.

## 5. Applications of Stable Isotopes (C, H, S) in Shales and Crude Oils

Hydrogen isotopes are used occasionally in petroleum exploration to support biomarkers data but are widely used in gas exploration, while sulfur isotopes are rarely used in petroleum exploration. However, carbon isotopes are by far the most frequently used in gas and petroleum exploration. The following sections describe the uses of the isotopic compositions of carbon, hydrogen, and sulfur in petroleum and gas exploration.

### 5.1. Stable Carbon Isotope

**Figure 2:**
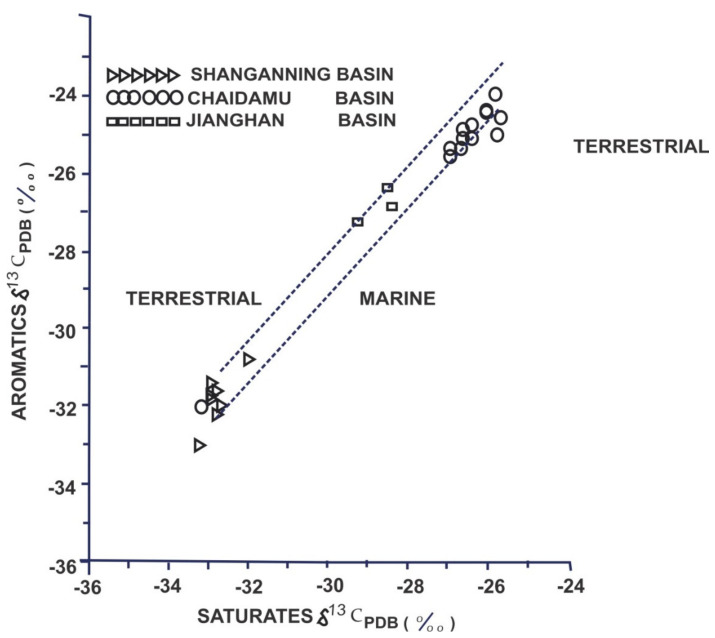
A plot of δ^13^C saturates versus δ^13^C aromatics (Reproduced from [91] with permission of Elsevier).

The organic materials in a source rock must be characterized to determine if a formation will generate oil, gas, or both. Visual characterization, extraction, and identification of specific biomarkers and stable isotopes are all techniques that can be used to define organic matter [85]. Craig [86] and Silverman [2,87] discovered that oils from terrigenous sources were more depleted in ^13^C than oils from marine sources in the early studies of stable isotopes in hydrocarbon exploration. Land plants were generally deficient in ^13^C compared to aquatic plants and marine heterotrophs, which appeared to mirror the trend seen in living systems. Organic matter in recent sediments has also been found to become depleted in ^13^C as the depositional facies shifts from marine to terrigenous [88]. Carbon isotopes are frequently utilized to distinguish between oils originating from marine and non-marine organic matter. The relationships between the isotopic composition of the saturate and aromatic fractions [1] reveal differences between marine and non-marine oils. The effects of the source on stable carbon isotope ratios have been examined [89] and it was found that as the concentration of terrestrial input increases, the δ^13^C values become less negative. In west Texas, isotopic age trends were employed successfully to correlate and differentiate crude oils on a geographical basis [4]. Moreover, a general trend of lighter isotope enrichment with the increasing geological age of oils was observed, possibly caused by variations in photosynthesis intensity and changes in the isotopic composition of atmospheric CO_2_ [50,90]. This observation formed the basis for isotopic age-dating methods developed later [88]. By fractionating the oil into saturates, aromatics, NSO (nitrogen, sulfur, oxygen), and asphaltene fractions, the usage of bulk isotopes can be taken a step further to improve the precision. Individual fraction isotope data can then be plotted in a variety of ways. As shown in Figure 2, a plot of saturates vs. aromatics can be very useful for grouping oils formed from comparable source materials or distinguishing oils from distinct depositional settings [91]. 

**Figure 3:**
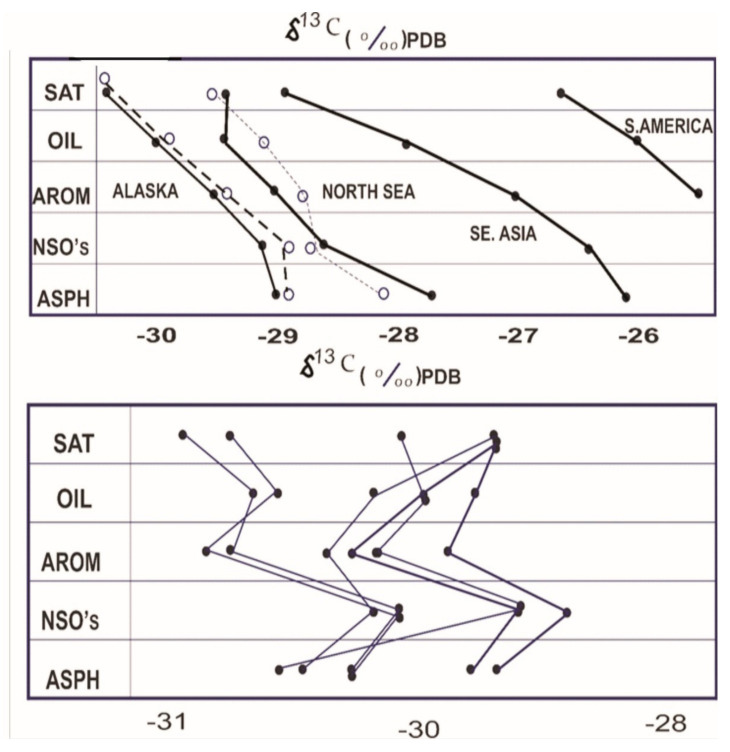
The isotopic relationship between the carbon isotope values of the various fractions and the source rock extracts. SAT: saturate hydrocarbons; OIL: whole oil; AROM: aromatic hydrocarbons; NSO’s: nitrogen, sulfur, oxygen compounds; ASPH: asphaltenes (Reproduced from [50] with permission from Elsevier).

The Stahl-type curves [92] can also be constructed using the isotope values. The stable isotope value for the kerogen can be integrated into this graph if the suspected source rock is available [21]. If the correct source rock is chosen, the individual fractions will have a direct link, as shown in Figure 3. The advantage of this method is that ^13^C values distinguish the carbon in different fractions and provide a more powerful correlation tool, though caution must be exercised in distinguishing the effects of increasing maturity [93], phase effects, and subsequent reservoir changes such as water washing [94] or biodegradation [95]. Understanding and quantifying these effects is therefore critical for the reliability of isotope correlation [96,97]. As a result, the factors that can alter the isotopic composition of crude oils and source rocks will be discussed in the following sections. 

#### 5.1.1. Source and Depositional Facies

Crude oil source rocks are generated from a variety of source materials that are deposited in a variety of depositional conditions [98]. Many factors will impact the isotopic compositions of these source materials, such as plants, phytoplankton, and bacteria, when they are first generated through photosynthesis. All of the original isotopic source signals will be homogenized and change slightly as this organic material is buried, degraded, and eventually heated to generate crude oils or natural gas [21]. However, because different crude oils are generated from diverse mixtures of source materials, some variations in the eventual isotopic composition of the producing crude oils are reasonable to expect. As a result, the isotopic composition of various crude oils from various sources is used. 

As stated by the basic reaction below, the starting point in the generation of crude oil in atmospheric CO_2_ begins through the process of photosynthesis, which leads to the incorporation of that C into the cell wall components, providing energy to promote plant growth.

6CO_2_ + 12H_2_O ⟶ C_6_H_12_O_6_ + 6H_2_O + 6O_2_

The organic material from living plants and aquatic organisms is deposited in a variety of environments after they die. A substantial portion of this material will deteriorate depending on the environment, but a small part will be absorbed into the sedimentary record, go through diagenetic processes, and then thermally transform the residual organic matter to generate oil, gas, or both [21,98]. The level of fractionation between atmospheric ^13^CO_2_ and ^12^CO_2_ during photosynthesis varies depending on whether the source material is a land plant or a marine organism, and the extent of fractionation within land plants is further determined by distinct photosynthetic cycles.

The isotopic compositions of plants that were initially determined [86] showed that most of the plant materials investigated at the time had a rather stable δ^13^C value, about—27‰ in a thorough investigation on the carbon isotopic content of natural materials. It was noted that there did not appear to be any significant species or geographic effects; however, one grass with a δ^13C^ value of 12‰ was later identified as a C_4_ plant. C_3_ plants, as defined by [86,99], incorporate CO_2_ from the atmosphere through carboxylation of ribulose bisphosphate. CO_2_ is absorbed by C_4_ plants by the carboxylation of phosphoenolpyruvate. The carboxylation product is transferred from photosynthetic cells’ outer layer (mesophyll cells) to the inner layer (the bundle sheath), where ribulose bisphosphate carboxylase decarboxylates and refixes it. C_4_ plants have lower negative δ^13^C values than C_3_ plants, according to isotope research. Plants with the C_3_ cycle, such as grasses, corn, and maize, grow in cooler, wetter climates and have isotope values in the 26 to 30 per mil range. Plants with the C_4_ cycle, such as grasses, corn, and maize, grow in dryer, hotter climates and have isotope values in the 10 to 14 range. The difference in the isotopic composition of C_3_ and C_4_ plants has become one of the primary methods for distinguishing these plant kinds [100], and it also serves as a useful diagnostic tool for distinguishing specific source materials. 

Due to variations in the isotopic composition of carbon sources, numerous investigations of marine and terrestrial plants have demonstrated a depletion of ^13^C in land plants compared to marine plants [32,86]. Isotopic differences similar to this have been observed in recent sediments [13,53,54,101]. In comparison to local marine fauna and flora, organic matter in recent marine sediments is often depleted in ^13^C [102,103,104]. Diagenetic factors may be the cause of the ^13^C depletion. The ^13^C depletion has been attributed to the diagenetic loss of ^13^C-enriched carboxyl carbon [105,106]. Alternatively, it was proposed that the maturation of ^13^C-depleted lipids in marine sediments results in the formation of ^13^C-depleted organic matter [104]. However, selective preservation, in which ^13^C-depleted refractory components of algae are selectively accumulated in sediment and the ^13^C-enriched components are microbially degraded and lost, could explain the ^13^C depletion of marine sedimentary carbon [107]. Because source rocks are heterogeneous mixtures of many different types of organic material, the resulting isotopic composition of the source rock will be a weighted average of the isotopic compositions of all the different preserved and residual source materials, and thus the situation becomes far more complicated and less specific. As a result, depending on the initial mixture of source materials, there will be changes in the bulk isotopic compositions of the source rocks, and hence the oils. The significant contribution of terrestrial organic debris to the organic precursors of marine organic matter has been widely thought to explain the relative depletion in ^13^C of several marine sediment samples [2,5,108]. One explanation for differences in the resulting crude oils is isotopic variations in the source materials. 

**Figure 4:**
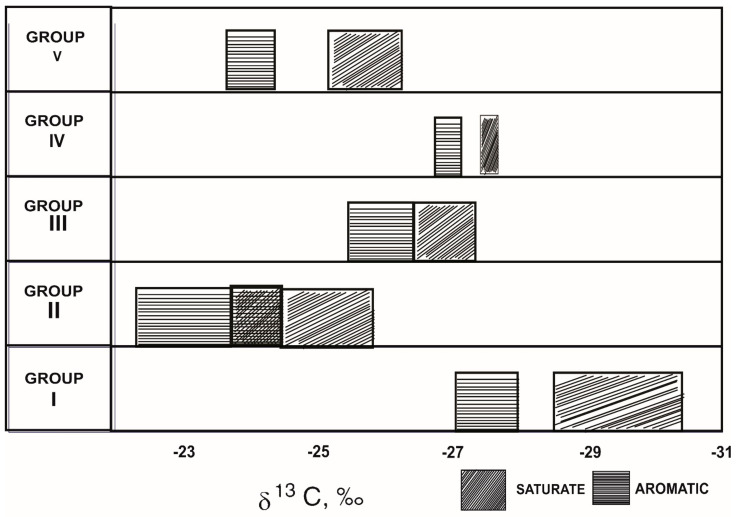
Saturate and aromatic carbon isotopic data of the crude oils from Brazil. (Reproduced from [109] with permission from Elsevier).

Moreover, there exists a comprehensive geochemical analysis of oils from offshore Brazil, using a variety of characteristics to distinguish oils from distinct depositional settings [109]. Based on a variety of criteria, including δ^13^C values, the oils were categorized into five groups (I to V). Table 2 summarizes some of the bulk parameters for each category of oils, including bulk isotope analyses, elemental analyses, and alkane distributions. Figure 4 shows how the oils were grouped into five types using δ^13^C values of the saturated and aromatic hydrocarbons. Group II oils are isotopically heavy (δ^13^C values about 25‰), which could be explained by increased salinity. Carbonate complexes are preferred as a carbon source for photosynthesis by plants from saline environments. These have a higher ^13^C content than atmospheric carbon dioxide, which has a higher ^12^C content [110].

#### 5.1.2. Biodegradation

Biodegradation has a well-defined set of changes that occur during the degradation process [85]. The primary changes in isotopic composition, on the other hand, are due to bulk changes in the oil rather than changes in individual molecules. The biodegradation of crude oil in the reservoir is a significant secondary alteration process with significant economic implications. Although the actual mechanisms involved in the in-reservoir biodegradation of crude oil are still being studied (e.g., site and rate of degradation, nutrient availability, and nature of by-products), their impact on the composition and physical qualities is well documented [111]. In most cases, the components of crude oils are removed sequentially in the order of n-alkanes > monocyclic alkanes > alkyl benzenes > isoprenoid alkanes > alkyl naphthalenes > bicyclic alkanes > steranes > hopanes. Many compound-related markers for determining the level of crude oil biodegradation have been proposed based on this sequence [112]. Biodegradation’s effects on the isotopic compositions of individual compounds have been explored for prospective use in petroleum and environmental research [57,113,114,115,116,117,118,119,120,121]. The effects of slight and moderate biodegradation on light hydrocarbons (C_5_–C_9_) consistently lead to ^13^C enrichment for each surviving molecule, according to studies by [57,119]. However, slight and moderate biodegradation resulted in insignificant enrichment in residual C_10_–C_14_ n-alkanes, with a maximum δ^13^C increase of 0.5‰, according to [111,114]. The stable carbon isotope compositions of natural gas components (C_2_–C_5_) are significantly fractionated during biodegradation, as has long been observed [122]. During biodegradation, lower carbon number molecules are removed first, followed by higher carbon number ones. Except in extreme cases of biodegradation, the amount of change in the aromatic and polar fractions is negligible [1,13]. The impact on the isotopic composition of the saturate fraction, on the other hand, can be much greater because n-alkanes make up a large portion of the saturate fraction, and their removal alters the isotopic composition, causing the saturate fraction to become isotopically heavier in general, as shown in the Stahl diagram in Figure 3. 

#### 5.1.3. Maturity

**Figure 5:**
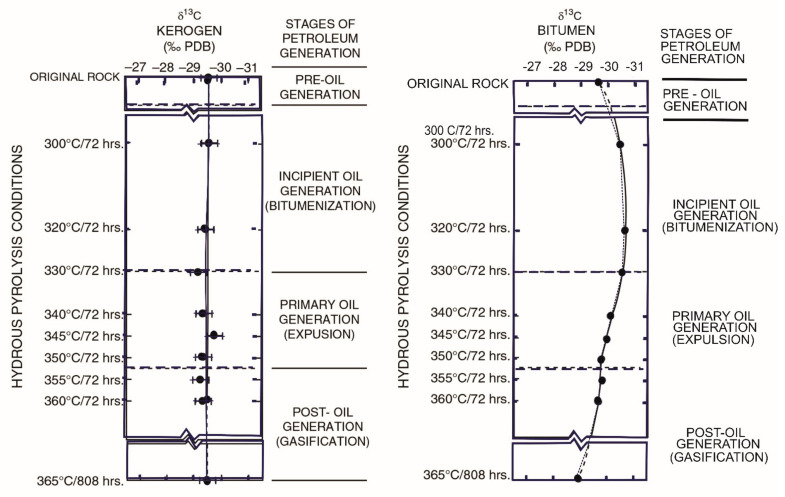
Changes in the isotopic compositions of crude oils and kerogen with maturation. (Reproduced from [124] with permission from Elsevier).

Unlike biodegradation, maturity can have an impact on the isotopic composition of all fractions, as maturity causes carbon–carbon bonds to break in all fractions, resulting in increased proportions of lighter hydrocarbons. Crude oil is formed by burial and maturity, and as a result of thermal maturation, there will be some variation in isotope composition. The effects of maturation on the ^13^C values of kerogens in the natural system have been thoroughly studied [123,124]. The kinetic isotope effect dominates during the maturation phase, resulting in the preferential breaking of bonds containing the lighter isotope. As a result, the heavier isotope will be enhanced in the residual fractions, and the overall trend will be one of isotopic enrichment as maturity increases. The degree of enrichment will differ from one oil to the next and will also be determined by the level of maturation. Oil-to-gas cracking degrades oil by turning it into low molecular weight molecules in a reservoir. Isotopic effects are more likely to represent kinetic isotope fractionation in this scenario, as ^12^C–^12^C bonds are preferentially cleaved over ^13^C–^12^C bonds, leaving the residual oil enriched in ^13^C [96,97]. The residual kerogen will become isotopically heavier with increasing maturity of source rocks, and the cracking of the kerogen within the source rock to generate oil will have similar effects. The products formed from kerogen will initially be isotopically light, but as maturity levels rise and more isotopically enriched kerogen is thermally degraded, the resulting products will become isotopically heavier. In general, kinetic isotopic fractionation in processes involving compounds with six or more carbon atoms is insignificant [96,97]. An additional isotopic fractionation step must be considered for condensates or light oils. As the subsurface gas phase (“gas condensate”) is raised to the surface and temperature and pressure decrease, condensates (usually C_6+_ compounds) condense from the subsurface gas phase (“gas condensate”). Because we are dealing with a transition from a liquid state (“oil”) to a vapor state (“condensate”), isotope effects will be limited during this process. Any isotope effect will be due to equilibrium isotope effects rather than the kinetic isotope effect in this case. However, in comparison to the oil from which they emerged, condensates will preferentially accumulate smaller molecular weight molecules. Differences in bulk δ^13^C between low and high molecular weight molecules will be reflected in bulk δ^13^C values for oil and condensate [96,97]. There are two ways to look into isotope effects that occur during the maturation and generation process. First, accumulated oils derived from a single source rock but maturing at distinct rates based on biomarker maturity characteristics can be isotopically identified, with discrepancies in the δ^13^C values attributed to maturation. This also presupposes that no further in-reservoir changes have happened. Laboratory maturation investigations, such as hydrous pyrolysis experiments [125], where an immature source rock sample is heated in the presence of liquid water at subcritical temperatures (<374 °C), provide an alternative option. Although these tests use relatively high temperatures (300–365 °C) to compensate for the long period required to generate oil in the natural system (106–109 years), the pyrolysates released from potential source rocks are similar to natural crude oil [125]. The vitrinite reflectance and atomic H/to C ratio of pyrolysates, which were measured in the matured source rocks, revealed similar alterations to those found in the natural system [124,126]. Immature source rock samples from the Woodford Shale were artificially matured to see how thermal maturation affected the δ^13^C values of kerogen, bitumen, and crude oil. It was discovered that as thermal maturation increased, the expelled oil became more enriched in ^13^C. (Figure 5). As stated above, the increased cleavage of ^13^C–^12^C bands in the bitumen as it continues to decompose into expelled oil and preferential cleavage of ^12^C–^12^C bonds in the expelled pyrolysate as it decomposes into gases and pyrobitumen can be attributed to the kinetic isotope effect and increased cleavage of ^13^C–^12^C bands in the bitumen as it continues to decompose [21].

#### 5.1.4. Age Dating

**Figure 6:**
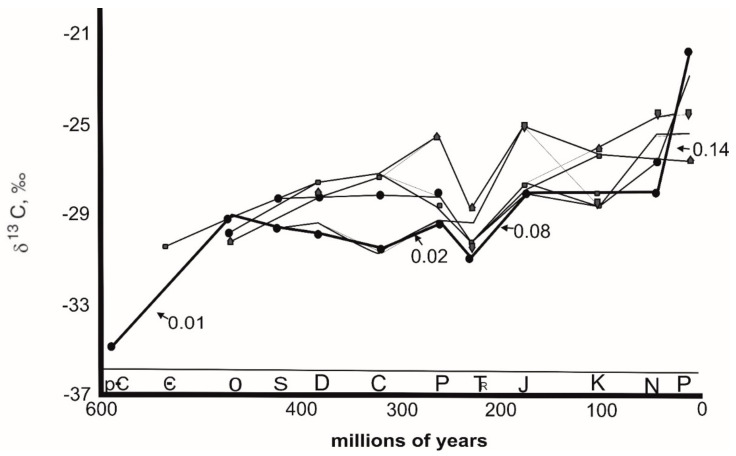
Stable carbon isotope composition of crude oils versus geologic age (Reproduced. from [88] with permission from Elsevier).

To age date oil samples, exploration studies have used several biomarker parameters that can be linked to certain evolutionary processes [127]. The interest in age dating stems from the fact that in many exploratory cases, only oil samples are recovered, and being able to anticipate the age of the source rock from crude oil properties is extremely important in determining the source formation responsible for the development of a certain oil. Andrusevich et al. [88] studied the possibility of age dating crude oils using stable carbon isotope compositions of different fractions of oil samples and found that the oils became more enriched in ^13^C as geologic age decreased (Figure 6). Changes in δ^13^C, in particular, are linked to global fluctuations in ^13^C carbonate carbon [88]. Previous studies of the stable carbon isotopic composition of oils (δ^13^C) over geologic time have found a general tendency of ^13^C enrichment with decreasing age [20,128]. The δ^13^C value of oil is often determined by the δ^13^C value of the kerogen in the source rock from which it was derived. The δ^13^C value of kerogen is determined by the sorts of organisms maintained as well as the δ^13^C values of its substrate, which leads back to the original photosynthetic conditions as stated above. Assuming that the present is the key to the past, variables known to be essential in the modern biosphere, such as temperature, pCO_2_, and depositional settings, are likely to have influenced the stable isotope composition of carbon in ancient biospheres. Organic carbon and carbonate carbon production, as well as their corresponding δ^13^C values, would have been influenced by changes in these settings [129]. The geologic record includes changes in the relative quantities of atmospheric gases such as CO_2_ and O_2_ as well as fluctuations in the δ^13^C values of organic and carbonate carbon [21,129]. Variations in carbon isotope values over time can be explained very readily. The first CO_2_ in the atmosphere came from the outgassing of the core and had a fixed isotopic composition [21,128]. The CO_2_ content of the atmosphere declined over time as more complex photosynthetic creatures and plants evolved, and the atmosphere became isotopically heavier in general [20,21,128]. There were isotopic excursions where CO_2_ became even heavier as a result of considerable productivity increases, and the principal photosynthesizers took on a heavier isotopic signature as a result. Because most fossil fuels and their combustion products have isotopic values in the range of 25 to 35 per mil [21], the isotopic composition of atmospheric CO_2_ is becoming lighter as the burning of fossil fuels increases. 

#### 5.1.5. Carbon CSIA

**Figure 7:**
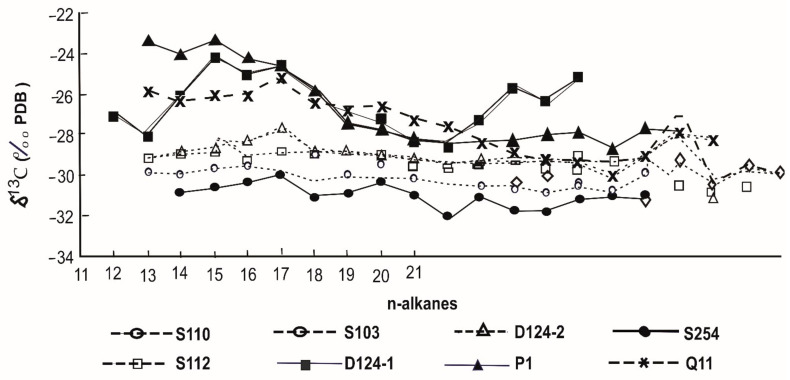
Carbon isotopic compositions of individual n-alkanes in source rocks of the.Western depression of the Liaohe Basin (Reproduced from [151] with permission from Elsevier).

It took over two decades to develop the ability to determine the isotopic composition of individual compounds. Continuous flow determination of carbon isotope values of individual compounds in complex mixtures of geochemical interest is possible with gas chromatography–isotope ratio mass spectrometry (GC–IRMS) [130,131,132,133]. Only bulk isotopic data were available before the introduction of GC–IRMS [102], which lack a lot of specificity due to the heterogeneous character of the source material [134]. As a result, isotopic data from crude oils and source rock extracts must be interpreted in conjunction with other geochemical information [135]. The use of GC-IRMS now allows the isotopic composition of individual compounds in crude oils and source rock extracts to be determined. The n-alkanes and isoprenoids have received the greatest attention since they are often well resolved in chromatograms [136]. Individual n-alkane δ^13^C values have been widely employed in oil–oil and oil–source rock correlations [137,138,139] as well as reconstructions of the paleoenvironment and paleoclimate [132,140]. The isotopes of individual compounds provide an additional correlation parameter, as well as the ability to determine whether or not many compounds are derived from the same precursor molecule. This is especially true for the pristane to phytane ratio [141], which is one of the most extensively used geochemical parameters in geochemical investigations. This ratio is commonly utilized for correlation as well as indicating the depositional environments and redox conditions. However, this latter application assumes that both compounds are derived from the side chain of chlorophyll, and if they are not, the ratio is invalid. The only way to determine whether they are from the same source is by measuring the carbon isotope value of these substances. A carbon isotopic investigation of crude oils and the Messel shale was conducted [131], which demonstrated that the isotopic values in the oils were quite comparable, indicating that they came from the same source, but the values in the Messel extract were significantly different, indicating that the pristane and phytane came from distinct origins. Isotopic compositions of individual compounds have also been successfully used to characterize specific components in the saturated hydrocarbon fraction and as a parameter to establish oil–oil and oil–source rock correlations [53,54,137,142,143]. This technique has been used by several researchers to assess the presence of uncommon polycyclic alkanes in extracts of source rocks and crude oils where hopanoids and steranes were either absent or present in extremely low quantity [144,145,146,147,148]. The assumption behind the oil–source rock correlation is that the oil and the corresponding source rock have similar δ^13^C values. To use isotopic compositions from crude oils and source rock extracts for direct oil–oil and oil–source correlations, two pre-requisites must be met: first, the maturity level of the oil and corresponding source rock must be similar; and second, secondary alterations such as hydrocarbon expulsion, migration, biodegradation, and water washing, among others, must have had no significant effect on the isotopic composition of either the oil or potential source rock [149,150]. Regardless of whether bulk or compound-specific isotopes are being identified, these requirements must be met. Carbon isotope profiles of n-alkanes can easily distinguish source rock samples from different areas within a basin, as demonstrated by [151], who analyzed δ^13^C values of specific n-alkanes for different formations throughout the Liaohe Basin in China. The n-alkanes of one of the Es4 members have a relatively constant isotopic profile, with δ^13^C values ranging from 28 to 32‰, whereas the n-alkane δ^13^C values for the other Es3 source rocks range from 23 to 28‰, with a trend toward isotopically lighter values with increasing n-alkane chain length, typically 2–5‰ [152,153]. The n-alkanes found in the samples have similar characteristics to the Es4 source rocks, implying a saline lacustrine environment that is rather closed (Figure 7; [151]).

#### 5.1.6. Application of Stable Carbon Isotopes to Gas Exploration

**Figure 8:**
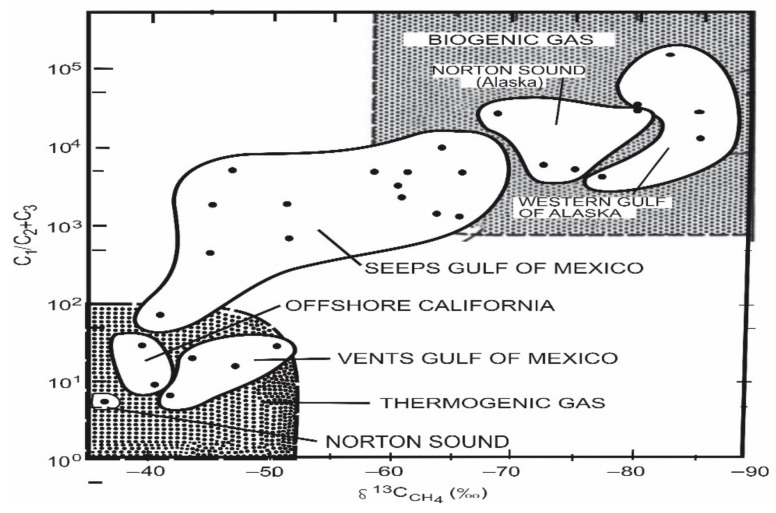
The relationship between carbon isotope values in methane and the C_1_/(C_2_+C_3_). compositional ratios of natural gas samples. (Reproduced from [154] with permission from Elsevier).

Natural gases, unlike crude oils, are generally simple mixtures, limiting the number of factors that can be used to differentiate gases from different sources [21]. Hydrocarbons in the C_1_ to C_5_ range will predominate in most natural gas samples, with varying quantities of CO_2_, H_2_S, and N_2_ [21]. The maturity of the source rock or oil from which the gas was formed will, to a significant extent, affect the proportions of individual components. At high levels of thermal maturity, methane-dominated gases are generated, whereas at low levels of thermal maturity, biogenic activity can result in methane-dominated gases. Differentiating biogenic from thermogenic methane is one of the more common uses of stable isotopes. Biogenic gas has a very light isotope, often around 70 per mil, whereas thermogenic methane has an isotope of 30 to 50 per mil [21,154]. This was realized many years ago, which led to one of the first correlation or source discrimination diagrams [154]. Essentially, this is a cross plot of methane δ^13^C vs. C_1_/C_2+_ ratio, and as seen below, biogenic samples are characterized by isotopically light methane and a high C_1_ to C_2+_ ratio (Figure 8). Thermogenic gas samples will be heavier isotopically and have lower compositional ratio values. There is also a mixed gas area were assigning a specific source to the gases in that region is a little more challenging. The kinetic isotope effect is responsible for the variance in these isotopic ratios [21,154]. Bacteria preferentially cleave ^12^C–^12^C bonds, resulting in the formation of isotopically light biogenic gas. From a thermogenic standpoint, these bonds take less energy to break at low levels of maturity than a ^12^C–^13^C bond. As the maturity level rises, more bonds containing the heavier isotope will be broken, causing the methane produced to become heavier isotopically. Natural gas samples have fewer alternatives for correlation or discrimination than crude oil samples, as previously stated [21,154]. The isotopic composition of the individual compounds in the gas samples, as well as variations in the relative quantities of the individual compounds, must be used to make these associations [21,155]. The GC-IRMS technique has been shown to be quite beneficial, making it comparatively simple to determine the isotopic compositions of individual compounds in complex mixtures. Before the invention of the GC-IRMS, each compound had to be physically isolated to determine its isotopic composition. 

**Figure 9:**
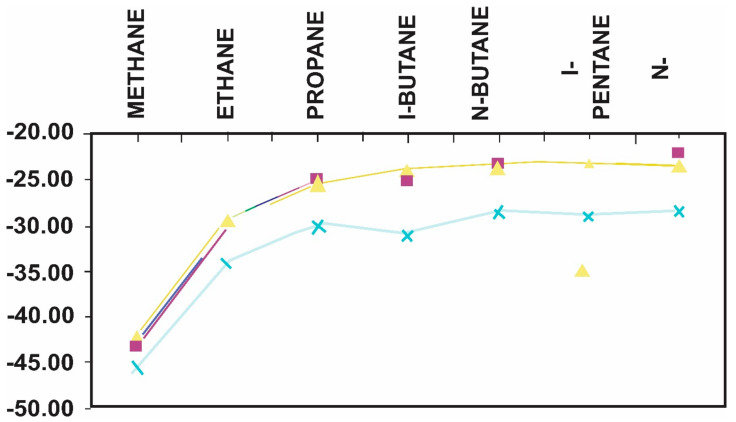
Diagram establishing the relationships between families of gases within a specific. Basin (after [21]).

With the GC-IRMS system, it is of course possible to simply introduce the sample into the GC and obtain the isotopic composition of each component in a relatively short period. It is also worth noting that these individual compounds can have both carbon and hydrogen isotope values, and a combination of the two isotopes offers an extra correlation parameter [21,154,155]. Simply plotting the isotope levels of specific compounds against their carbon number is the most useful application of isotopes for correlation purposes. Then, with a sufficient number of gas samples from the same basin, those that are connected will plot together, but those from other sources or created at different levels of maturity will plot independently, as illustrated in Figure 9 [21].

**Figure 10:**
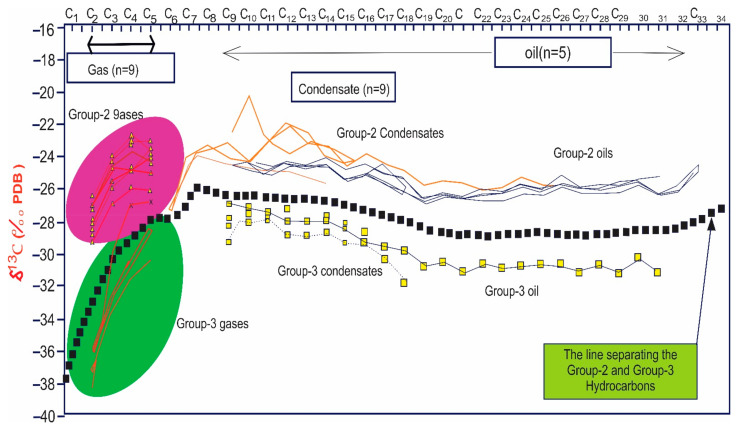
Plot showing the relationships between gases and condensates produced from the same formation (Reproduced from [155] with permission from Elsevier).

This graph has many interesting extensions that can reveal a significant amount of additional information. As previously stated, we are constrained in our ability to distinguish between natural gas sources. However, after examining the isotopic composition of the individual compounds in the condensates, it has been proven that for wells that produce both gas and condensates, the plots presented above can be extended into the condensate range. The isotope values for the individual compound in the condensate will simply be an extension of the relevant gas sample, as illustrated if the condensate and gas are generated from the same source and at the same maturity level (Figure 10) [155]. The benefit of collecting these extended plots is that we can utilize the isotopes to establish the relationship between gas and condensate, but there are also biomarkers, such as diamondoids, in the condensates that may be used to correlate or discriminate between samples [21,155]. 

### 5.2. Stable Hydrogen Isotope

Hydrogen isotope has been applied to oil–oil and oil–source rock correlations in petroleum geochemistry [18,156]. The δD signature of the precursor must be conserved throughout sedimentation, burial, diagenesis, and catagenesis for the stable hydrogen isotopic composition (δD) of sedimentary hydrocarbons to resemble that of their biosynthetic precursors [10]. Over long periods of geological time (millions of years), diagenetic and catagenetic effects are thought to induce considerable hydrogen (H/D) exchange between organic hydrogen and hydrogen species in the surrounding environment [9,14,19,157,158,159,160,161,162,163]. Thermal maturation, in particular, has been discovered to play an important role in the change of indigenous δD signatures, with increasing maturity resulting in a general enrichment of D in hydrocarbon fractions and individual hydrocarbons [14,19,162,163]. The hydrocarbons are expected to be exchanging hydrogen with the comparatively D-rich formation fluids [158,164]. The influence of maturation on the δD values of sedimentary hydrocarbons (n-alkanes, pristane, and phytane) in a sequence of marine source rocks from the Perth Basin was demonstrated by [162] (Western Australia). Immature source rocks (%R_0_ = 0.53) have distinct δD signatures, with pristane and phytane considerably reduced in deuterium (D) compared to the n-alkanes. The discrepancy between the δD values of n-alkanes and isoprenoids gradually reduces as maturity increases (up to %R_0_ = 1.13). Pristane and phytane become increasingly D-enriched, while then-alkanes maintain a steady isotopic composition until late maturity when D-enrichment in n-alkanes becomes considerable. Isotopic exchange processes linked with thermal maturation have been attributed to the D enrichment.

A similar trend of D enrichment in n-alkanes and isoprenoids was observed in other studies [14,163]. The former study presented the effects of thermal maturity on n-alkane and isoprenoids δD levels in two sediment sections in Poland (Kupferschiefer, KS) and Germany (Posidonia Shale). With increasing age, all n-alkanes and isoprenoids were enriched in D, with isoprenoids enriching at a faster rate than n-alkanes. The latter investigated a 450-meter core of Early Cretaceous lacustrine sediments from West Africa, which ranged in maturity from immature to early mature (%R_0_ 0.55–0.7). The effects of maturation on the δD values of individual sedimentary hydrocarbons from sedimentary sequences with a wider maturity range (%R_0_ 0.6–1.6), such as Paqualin-1 and Vulcan-1B from the Vulcan Sub-basin offshore northern Australia has been analyzed [10]. The enrichment in D in isoprenoids was found to be connected to the epimerization of pristane and phytane and to correspond substantially with traditional maturity criteria. D enrichment was seen in pristane and phytane isolated from post-mature Paqualin-1 sediment, demonstrating that D enrichment was maintained at extremely high maturity, more so for regular isoprenoids than n-alkanes. This corroborated the theory that hydrogen (H/D) exchange, rather than initiating free radical hydrogen transfer, causes the observed shift in δD values. In the sedimentary environment, a mechanism was postulated to account for both H/D exchange and the epimerization of pristane and phytane. Pristane was found to be more enriched in D than phytane across the Vulcan Sub-basin sequences, indicating that they exchanged hydrogen at equal rates during maturation. This was attributed to a reduced relative algal input to the isoprenoids [10].

#### 5.2.1. Hydrogen CSIA

δD measurements of bulk OM or whole fractions from crude oils or sediment extracts were the only way to analyze the distribution of stable hydrogen isotopes in sedimentary OM. Because aliphatic C-bound hydrogen is probably the most isotopically conservative, isotopic analysis of individual aliphatic compounds with solely C-bound hydrogen (e.g., n-alkanes) is appealing. Compound-specific hydrogen isotope analysis [18,165] has made it possible to determine the D/H composition of individual compounds in complex mixtures. Because it has the highest mass difference between its two stable isotopes (D and H), and hence the greatest natural variability in stable isotope ratios of all elements, hydrogen compound-specific isotope analysis has shown remarkable promise in petroleum geochemistry. The existing literature has looked into the relationship between δD values of whole crude oils and bitumen sand and their origins (including organic matter type and depositional circumstances), thermal maturity, and secondary processes such as biodegradation [147], mixing [10], and migration [147]. In a different study, it was explained how different organisms fractionate hydrogen isotopes in the production of lipids [166] while another one reported individual n-alkanes and isoprenoids δD values as evidence of large and rapid climate change [9]. Furthermore, the efficacy of hydrogen CSIA in petroleum correlation studies using a large number of crude oil samples from the Western Canada Sedimentary Basin was illustrated [10]. Additionally, it was shown that sedimentary aliphatic hydrocarbon D values can be utilized to determine the age of source rocks and crude oils from the Perth Basin (WA) and Vulcan Sub-basin (Timor Sea) [9,147,166,167]. Pristane (Pr) and phytane (Ph) have substantially lower δD values than n-alkanes; however, this difference diminishes with age due to thermal hydrogen isotope exchange. These studies suggested that the δD measurements of sedimentary hydrocarbons are a good source of the effects-aware maturity metric. For Devonian source rocks from the Western Canada Sedimentary Basin (WCSB), the D/H of biomarkers has been employed as a maturity proxy [147]. Additionally, how various organisms fractionate hydrogen isotopes in lipid production was investigated [168]. The δD values of compound-specific lipid biomarkers extracted from peat deposits [15] and sediments [16] have been utilized as a proxy for palaeoenvironmental and palaeoclimatic conditions. During the Messinian salinity crisis, δD values of individual n-alkanes and isoprenoids were reported as evidence of substantial and rapid climatic fluctuation [147]. Using crude oil samples from the Western Canada Sedimentary Basin, the utility of hydrogen CSIA in petroleum correlation studies and palaeoenvironmental reconstructions was evaluated [169]. Next, the ability of lipid δD values to be preserved in Miocene lacustrine sediments and plant fossils from Clarkia, Idaho, USA was demonstrated [170]. The Clarkia sediments they looked at are the oldest samples known (15–20 million years) with original δD values that appear to have been retained. There is concern that long-term diagenetic effects can result in considerable hydrogen isotope exchange between organic hydrogen and the surrounding environment [157,158,159,160,161,171]. Thus, the extent to which hydrogen CSIA can be employed for palaeoenvironmental investigations, particularly when applied to older sedimentary organic matter, is of interest. Organic geochemists and palaeoclimatologists are interested in the δD values of organic compounds preserved in sediments because they can represent the isotopic composition of water in ancient environments. Because water mobility and the energy transferred as it changes physical states are major elements in weather and climate, hydrogen isotopic fractionations are assumed to be linked to a range of naturally occurring events in the hydrological cycle. ‘Meteoric’ waters are those that have gone through the hydrological cycle. Temperature, altitude, latitude, closeness to the ocean, and other factors affect the isotopic values of meteoric waters [50,172]. Water is the primary hydrogen source for photosynthetic organisms, and the deuterium concentration in the source water is mirrored in the deuterium composition of the organism [168]. The remains of species are eventually absorbed into sediments and added to petroleum’s organic matter. Similarly, CSIA of hydrogen to investigate Late Carboniferous to Late Permian torbanites from Torbane Hill, Scotland, and the southern hemisphere, eastern Australia, was used [17], where it was discovered that the δD values of n-alkanes and isoprenoids were similar to those found in modern biological samples, which implies that their indigenous δD signatures may have been preserved for at least 260–280 million years.

#### 5.2.2. Application of Hydrogen Isotope to Gas Exploration

The hydrogen isotopes of alkane gases, as well as other saturated and aromatic hydrocarbons combined with carbon isotopes, play a critical role in the identification of natural gas genetic-type analysis, parent material source, maturity, mixing, biodegradation, and (TSR) [147,173,174,175,176,177,178,179,180,181,182,183,184,185,186,187,188]. Apart from the parent material type, maturity, biodegradation, and TSR, the water environment during the deposition and diagenesis of source rock (such as salinity) also plays a crucial effect [147,189]. Many studies have been conducted on the hydrogen isotopic characteristics of natural gases from the Ordos Basin Permian and the Upper Triassic Xujiahe Formation of the Sichuan Basin; the origin and source of natural gases have also been analyzed based on the carbon and hydrogen isotopic and chemical composition characteristics of the alkane gas. The natural gases in the above two places have been proven to be coal-formed gas [190,191,192,193,194,195,196,197,198,199,200], and hydrogen isotopic indices for detecting natural gas genesis and vitrinite reflectance (Ro) values have been proposed [147,197]. Some studies on factors affecting hydrogen isotopes of alkane gas have been conducted [167,197], but most of them focused on the composition of hydrogen isotopes and affecting factors of Permian natural gases (methane and its homologs) and the Upper Triassic Xujiahe Formation in the two basins from a single basin perspective, and a few made comparisons [197], but only for methane hydrogen isotopes. The composition, influencing factors, maturity, and natural gas genetic identity indicators of hydrogen isotopes of heavy alkane gases, such as ethane and propane, received little attention. In a recent study [201] the hydrogen isotopic compositions of coal-formed Permian gas in the Ordos Basin and Upper Triassic gas in the Sichuan Basin, and the hydrogen isotopic compositions of alkane gas in various areas were also studied. To develop and improve the coal-formed gas theory and genetic identification theory of natural gas, as well as to guide natural gas exploration, the influencing factors and their degree of influence on the hydrogen isotopic composition of alkane gases were investigated, and hydrogen isotope indexes of alkane gas capable of identifying the natural gas genetic type and Ro value of gas were proposed.

### 5.3. Stable Sulfur Isotopes

Sulfur isotopes have been utilized to correlate source rocks and oils in a rapidly buried basin that have not been altered by thermochemical sulfate reduction (TSR) [11,12,202,203,204]. Hydrocarbons are produced quickly in such a basin, and peak oil is anticipated to occur under semi-closed to closed circumstances. In case studies and experimental simulations, this property, together with strong H_2_S solubility and quick sulfur isotope homogenization, is thought to result in modest discrepancies (up to 2) in ^34^S values between mature kerogens and their generated oils [12]. With the δ^34^S values reported in closed range in both the oils and the source rocks, sulfur isotopes have been effectively used to correlate Cambrian-derived oils with Cambrian source rocks [11,12]. To identify the source of oils and oil-source rock correlations in oils and source rocks from the Tazhong area, Tarim Basin, China, bulk and individual n-alkane δ^13^C and individual alkyldibenzothiophene δ^34^S values were used [205]. The majority of the oils from the Tazhong area were most likely generated from Cambrian source rocks, and δ^13^C and δ^34^S values can be utilized to indicate the oil–source rock relationship in the Tazhong uplift, Tarim Basin, China. Sulfur isotopes have also been successfully used for oil–oil correlations in the oils from the Williston Basin of North Dakota and Saskatchewan [202]. The sulfur isotopic composition can be utilized to reconstruct changes in the sedimentary environment since large cycles between sulfate and sulfide are associated with organic and inorganic carbon and iron cycles [205,206,207]. A series of geological and geochemical studies, including a pyrite sulfur isotopic investigation of lacustrine black shales from the Yanchang Formation in Ordos Basin, China was recently carried out [208]. The paleoredox chemical conditions were confirmed to be the trigger for the anomalous sulfur isotopic compositions based on the analysis of sulfur isotope fractionation during the sulfur cycle.

## 6. Conclusions

This paper critically reviewed the early and recent trends in stable isotope geochemistry of organic elements in shales and crude oils. The bulk and compound-specific stable isotopes of H, C, and S, as well as their uses as source facies, depositional environments, biodegradation, thermal maturity, geological age, and oil–oil and oil–source rock correlation studies, were discussed. The applications of the stable isotopes of H and C to gas exploration were also discussed. Then, the experimental and instrumental approaches in the stable isotopes of H, C, and S were discussed. This review showed that the stable isotopes of H, C, and S could be utilized to establish the origin, depositional conditions, and thermal maturity of organic matter of shales, as well as oil–oil and oil–source rock correlation studies, which are critical to hydrocarbon exploration. However, there is limited literature that discussed the sulfur isotopic compositions of individual biomarkers in crude oils, while papers on compound-specific isotopic analysis of individual biomarkers in shales are very rare in the literature. The reason is that these compounds are present in relatively low concentrations and generally cannot be seen on a regular gas chromatogram; hence, it is not possible to obtain isotopic compositions of individual biomarkers on a routine basis, especially sulfur. The need here is to develop more routine molecular sieving methods that can be used to isolate concentrates of the biomarkers and then determine the isotopic composition of these compounds and provide another fingerprinting tool for correlation purposes. Conclusively, emerging isotopic techniques such as laser microprobe, ion microprobe, and NanoSIMs, which can collect data on the surface, are recommended for further stable isotopic studies on shales and crude oils.

## Figures and Tables

**Table 1:** Isotopic ratios of stable isotopes [21].

Element	Notation	Ratio	Standard	Absolute Ratio
Hydrogen	δD	D/H(^2^H/^1^H)	SMOW	1.557 × 10^−4^
>Carbon	δ^13^C	^13^C/^12^C	PDB	1.122 × 10^−2^
Nitrogen	δ^15^N	^15^N/^14^N	Atmosphere	3.613 × 10^−3^
Oxygen	δ^18^O	^18^O/^16^O	SMOW, PDB	2.0052 × 10^−3^
Chlorine	δ^37^Cl	^37^Cl/^35^Cl	seawater	−0.31978
Sulfur	δ^34^S	^34^S/^32^S	CDT	4.43 × 10^−2^

SMOW = standard mean ocean water; PDB = Pee Dee belemnite; CDT = Cañon Diablo troilite.

**Table 2:** Bulk and elemental properties of Brazilian offshore oils (Reproduced from [109] with the permission of Elsevier).

Property	Group I	Group II	Group III	Group IV	Group V
δ^13^C‰	< −28.0	−23.0 to −25.6	−25.4 to −26.6	−26.8 to −27.6	−24.4, −25.1
% Sulphur	Low	Medium	High	High	Medium
V/Ni	Low	High/medium	Medium	High	High
%Saturates	High	High	Low/medium	Low/medium	High
n-Alkanes’ dominance	C_23_-C_25_	C_17_-C_21_	C_17_-C_19_	C_18_-C_20_	C_20_-C_22_
Odd/even	High	High	Low	Low	Low
Pristane/Phytane	High	High	Low	Low	Low
Inferred depositional environment	Lacustrine/freshwater	Lacustrine/saline water	Marine evaporitic	Marine carbonate	Marine deltaic

## Data Availability

Not applicable.
